# Correlating the Energetics and Atomic Motions of the Metal-Insulator Transition of M_1_ Vanadium Dioxide

**DOI:** 10.1038/srep26391

**Published:** 2016-05-23

**Authors:** Jamie M. Booth, Daniel W. Drumm, Phil S. Casey, Jackson S. Smith, Aaron J. Seeber, Suresh K. Bhargava, Salvy P. Russo

**Affiliations:** 1 Chemical and Quantum Physics, School of Science, RMIT University, Melbourne VIC 3001, Australia; 2 Australian Research Council Centre of Excellence for Nanoscale BioPhotonics, Applied Physics, School of Science, RMIT University, Melbourne 3001, VIC, Australia; 3 CSIRO Manufacturing, Clayton VIC 3168, Australia; 4 Centre for Advanced Materials and Industrial Chemistry, School of Science, RMIT University, Melbourne VIC 3001, Australia

## Abstract

Materials that undergo reversible metal-insulator transitions are obvious candidates for new generations of devices. For such potential to be realised, the underlying microscopic mechanisms of such transitions must be fully determined. In this work we probe the correlation between the energy landscape and electronic structure of the metal-insulator transition of vanadium dioxide and the atomic motions occurring using first principles calculations and high resolution X-ray diffraction. Calculations find an energy barrier between the high and low temperature phases corresponding to contraction followed by expansion of the distances between vanadium atoms on neighbouring sub-lattices. X-ray diffraction reveals anisotropic strain broadening in the low temperature structure’s crystal planes, however only for those with spacings affected by this compression/expansion. GW calculations reveal that traversing this barrier destabilises the bonding/anti-bonding splitting of the low temperature phase. This precise atomic description of the origin of the energy barrier separating the two structures will facilitate more precise control over the transition characteristics for new applications and devices.

The reversible phase transition of VO_2_ at ~340 K occurs between a low temperature, insulating monoclinic structure, and a high temperature, metallic tetragonal form^1^,^2^,^3^. The transition between between the insulating and metallic forms results in a switch from transparent to absorbing in the near infra-red^2^,^3^,^4^, which can occur on time-scales as low as femtoseconds when triggered by laser pumping^5^. While this transition was first identified by Morin in 1959^1^, and explored more thoroughly in the 1970s by authors such as Goodenough^6^, Pouget^7^ and Mott^2^, the last decade has seen an explosion of research into devices based upon this transition^8^,^9^,^10^,^11^,^12^. The trigger for this has in part been the maturation of fabrication procedures which allow nanostructures of vanadium dioxide to be grown and utilized^13^,^14^.

Much of the existing theoretical research has been devoted to answering the question of whether the insulating form of VO_2_ is a band- or a Mott-Hubbard insulator^2^,^3^,^15^,^16^, in an effort to determine the roles of both correlations and lattice symmetry breaking in the transition, with most work confirming that both effects are important^16^,^17^,^18^,^19^,^20^. However, a complete description of the interplay between the energetics, the atomic rearrangements and the electronic structure of VO_2_ as it transitions between the monoclinic and tetragonal structures has remained elusive. Device design and optimization requires detailed knowledge of the energy landscape across the transition with respect to changes in the lattice structures. This knowledge has become particularly important in recent years with the development of devices based upon the modulation of the metal-insulator transition of VO_2_ by inputting stress or strain^12^,^13^,^21^,^22^,^23^.

Evidence for a soft mode connecting the tetragonal to the monoclinic structures was found as far back as 1978 by Terauchi and Cohen^24^, who found a lattice instability at the *R* point of the tetragonal structure using diffuse X-ray scattering. Gervais and Kress^25^ used a shell model to calculate the phonon dispersion curves of the tetragonal form of VO_2_, and also found a softening of the lowest frequency mode at the *R* point.

Beginning with the work of Cavalleri *et al*.^5^ pump-probe measurements have shed considerable light on the lattice dynamics occurring across the transition. A structural “bottleneck” associated with the phonon connecting the monoclinic and tetragonal structures was observed upon hole photo-doping^26^, suggesting that the insulating phase depends significantly on the lattice potential, indicating band-like character. Kim *et al*.^27^ used pump-probe measurements in conjunction with X-ray diffraction and found that the sharp resonance corresponding to the monoclinic A_*g*_ peak disappears at the transition with lower energy and less intense tetragonal B_*g*_ resonances replacing them. Wall *et al*.^28^ also used pumping to modify the lattice local potential and examine its effects on the coherent phonon spectrum of VO_2_, as an example of the general applicability of the use of pumping to induce a change in lattice potential, which can be used to study relaxation processes. However, a theoretical description of the interplay between the lattice potential and the atomic and electronic structure has proven elusive.

The computational study of Zheng and Wagner^29^ utilised the Quantum Monte Carlo approach to show that the MIT is a direct consequence of the change in structure, that is the monoclinic structure is insulating, and the tetragonal form exhibits metallic behaviour. While sounding trivial, there has been some conjecture over the coincidence of the structural and electronic phase transitions^27^,^30^, which Zheng and Wagner, and also this work resolve. Chen *et al*.^31^ explored the properties of the parameter space spanned by the *β* angle and the tetragonal c-axis using the DFT+U approach^32^, and suggested that changing orbital occupancy is initially responsible for opening the band gap as a result of dimerisation, which is widened by a subsequent increase in the antiferroelectric distortion.

Thus what is missing from the literature as it currently stands is an exploration of the energy landscape of the structural phase transition with respect to the metal-insulator transition in terms of exactly what constitutes the separation between the two structures. The intent of this work is to utilize a comprehensive computational approach to determining the processes occurring during the metal-insulator and structural phase transitions, and to combine it with experimental data to confirm the predictions of our calculations. Specifically, the outstanding questions we seek to answer are: i) literature data suggests a latent heat of ~40 J/g for the transition^33^, to what does this energy barrier correspond? Which particular atomic motions give rise to this barrier, ii) if a minimum energy path can be mapped between the structures, and the aforementioned atomic displacements determined, what are the effects of these displacements on the electronic structure? Are the structural phase transitions and metal-insulator transitions necessarily coincident as suggested by Zheng and Wagner^29^?

We start by computing the lowest energy path between the structures using the nudged elastic band technique^34^ and density functional theory to determine this energy landscape. The DFT data reveal that in order for the structural transition to occur, the inter-vanadium spacing along the [110] or 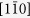 directions must be compressed, generating electronic repulsion and thus an energy barrier. High resolution X-ray diffraction measurements reveal anisotropic strain related to the atomic spacing in these directions in the monoclinic structure, which is not present in the tetragonal form. Frequency-dependent GW calculations reveal that the top of the barrier corresponds to the opening of the gap due to bonding/anti-bonding splitting as the vanadium atoms dimerise. The data indicate that the most efficient modulations of the transition temperature involve stress input along [110] or 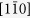 of the tetragonal structure or the [011] or 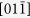 directions of the monoclinic structure, consistent with the action of doping with tungsten^35^.

## Results

### Structural Rearrangements

**Figure 1:**
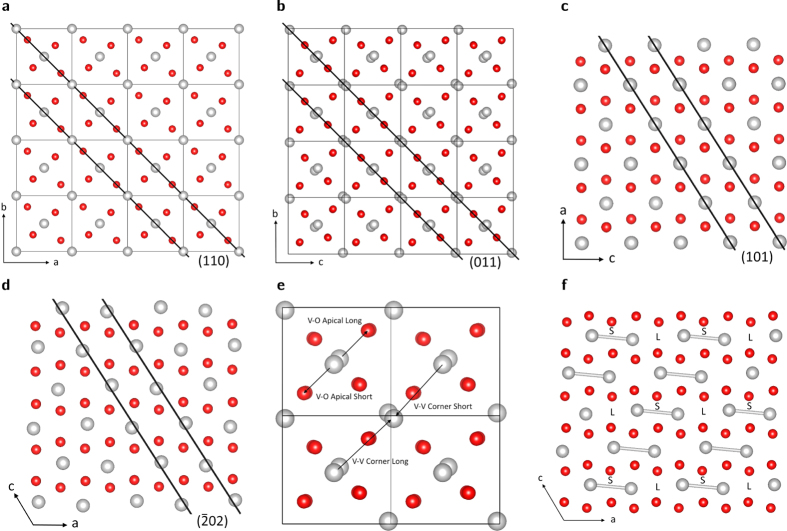
(**a**) View down 〈001〉 of the tetragonal structure, the (110) planes are indicated by black lines, (**b**) view down 〈100〉 M_1_ structure, the (011) planes are indicated by black lines, illustrating that they correspond to the same distance as the tetragonal (110) planes, the off-centre positioning of the vanadium atoms in the M_1_ structure from the anti-ferroelectric twist is also apparent (**c**) view down 〈010〉 of the tetragonal structure, the evenly spaced vanadium chains are visible, running parallel to the c-axis, and the (101) planes are indicated with black lines, (**d**) view down 〈010〉 of the M_1_ structure, with the Peierls paired vanadium chains visible running parallel to the a-axis and the 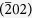 planes are marked with black lines. These are shifted by one half of a lattice spacing for better comparison to the tetragonal (101) planes, illustrating that both correspond to the same distance, (**e**) same perspective as (**b**) but with the “V-V Corner Long”, “V-V Corner Short”, “V-O Apical Long” and “V-O Apical Short” distances marked and (**f**) same perspective as (**d**) but with the “V-V Chain Short” and “V-V Chain Long” distances indicated by the letters “S” and “L” respectively.

The relevant structural characteristics of tetragonal and M_1_ VO_2_ are presented in Fig. 1. Figure 1a,b compare the view of the tetragonal structure down 〈001〉_*T*_ (the subscript T or M refers to tetragonal or monoclinic respectively) with the view of the M_1_ structure down 〈100〉_*M*_. The comparison illustrates that the structural rearrangements occurring in the transition from the tetragonal to the monoclinic structure orthogonal to the monoclinic a-axis can be summarized as an alternating off-set of the vanadium atoms from the centers of the oxygen octahedra. This off-set occurs in the long axis of the octahedra. The (110)_*T*_ and (011)_*M*_ planes of the tetragonal and monoclinic structure are also indicated with black lines, which reveals that they correspond to equivalent atomic spacings in each structure (although due to a slight expansion of the tetragonal structure its diffraction peak manifests at slightly lower angle).

Figure 1c,d present a comparison of the tetragonal and M_1_ structures down 〈010〉 (this axis is coincident for the tetragonal and monoclinic structures), which indicates that the changes occurring across the structural phase transition parallel to the monoclinic a-axis consist of the evenly spaced vanadium atoms (the “vanadium chains”) of the tetragonal structure pairing up (the so-called Peierls pairing), forming an alternating long-short pattern of inter-vanadium spacing. The (101)_*T*_ and 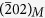 planes are indicated in the tetragonal and monoclinic structures respectively, the 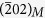 plane has been shifted by one half of its spacing to illustrate that it is the equivalent distance in the monoclinic structure of the (101)_*T*_ plane.

Figure 1e illustrates the four characteristic distances of interest in this study which are orthogonal to the monoclinic a-axis. The V-O Apical Long V-O Apical Short distances describe the amount to which the vanadium atoms are off-set from the center of the octahedron; if the numbers are equal then the atom sits at the center of the oxygen octahedron. The V-V Corner Long and Short distances describe the two shortest distances between the vanadium atoms on neighboring chains, these distances lie parallel to the (101)_*T*_ and (200)_*M*_, 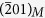 planes respectively. Figure 1f defines the V-V Chain Long (indicated by the letter “L”) and short (“S”) distances. These are the distances between the vanadium atoms in the chain which undergoes Peierls pairing. If both distances are equal, as in the tetragonal structure, the vanadium atoms are evenly spaced. As the tetragonal structure transforms into the monoclinic form, the atoms pair up and one of these distances decreases, while the other increases.

### Nudged Elastic Band Calculations

**Figure 2:**
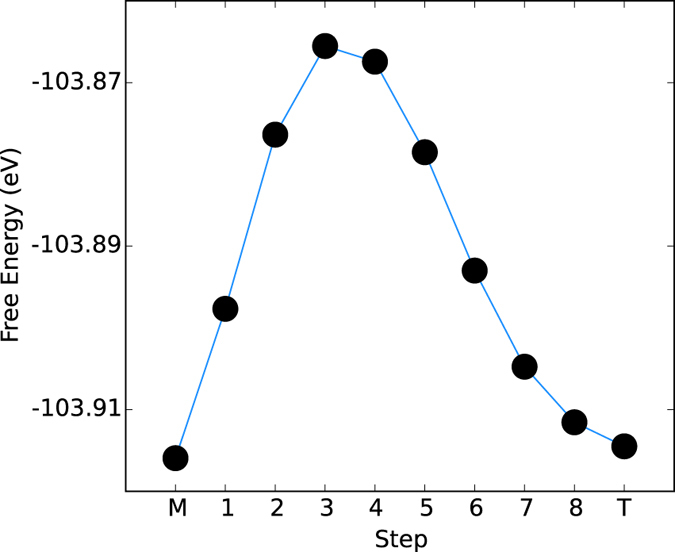
Total free energies (eV, black filled circles) of the structures across the transition from a combination of DFT geometry relaxations and the elastic band method. M and T correspond to the monoclinic and tetragonal structures respectively, while the intermediate structures are denoted by step numbers.

**Figure 3:**
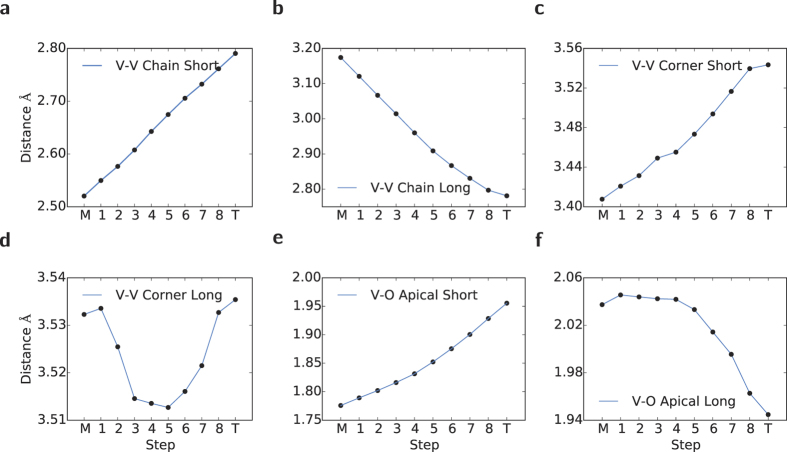
Variation of characteristic distances of the VO_2_ structure across the energy path determined by the elastic band calculations. The data points (filled black circles) are linearly interpolated to guide the eye. Of note are monotonic trends with step progression of all distances apart from V-V Corner long, which initially contracts, then expands. Illustrations of what each distance corresponds to are contained in Fig. 1.

The total free energies of the structures obtained along the minimum energy path between the monoclinic and tetragonal structures determined by the nudged elastic band method are plotted in Fig. 2. While the energies of the monoclinic and tetragonal structures are almost degenerate, there is a clear energy barrier between the two structures. This corresponds to an energy of 18.6 J/g, which compares favorably with the experimentally observed specific heat of the phase transition of ~40 J/g^33^. However, this result poses the obvious question: to what does this barrier correspond to in terms of the structural rearrangements? Figure 3a–f plot the a) V-V Chain Short (aka Peierls) spacing, b) the V-V Chain long distance, c) the V-V Corner Short distance, d) the V-V Corner Long distance, e) the V-O Apical Short and f) V-O Apical Long distance. Figure 3a,b indicate that the Peierls pairing distance increases continuously across the transition from monoclinic to tetragonal, while unsurprisingly, the corresponding long inter-vanadium distance decreases monotonically. This data simply expresses the fact that the evenly spaced vanadium atom chains running along the tetragonal c-axis (monoclinic a-axis) experience a Peierls distortion and adopt a long-short internuclear spacing configuration. The monotonicities of the plots do not suggest an origin for the energy barrier of Fig. 2.

Figure 3c,d however tell a different story. Figure 3c indicates that the short distance between the central vanadium atoms and the corner vanadium atoms (see Fig. 1c) increases monotonically across the transition, however the longer distance (plotted in Fig. 3d) initially contracts, and then expands. Comparison of Figs 2 and 3 suggest that the peak of the total energy corresponds approximately to the minimum in the long V-V corner distance. Figure 3e,f illustrate the trends of the apical vanadium-oxygen distances, and the shorter distance again displays a monotonic increase across the metal-insulator transition, however the longer distance initially plateaus, before decreasing significantly.

Thus the only behavior occurring across the metal-insulator transition consistent with an energy barrier, i.e. an initial increase and subsequent decrease, is the compression and expansion of the long V-V corner distance. This indicates that the force needed to effect the transition between the structures is directed approximately along the diagonals of the unit cell, perpendicular to the vanadium chains. This corresponds to the [110]_*T*_ and 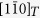 directions of the tetragonal structure. These directions describe the spacing of the {110} planes of the tetragonal structure, and the {011} planes of the monoclinic structure. Such an effect closely mirrors the observations of Pouget *et al*.^36^ who found that inputting a uniaxial stress along the [110]_*T*_ direction resulting in the appearance of the M_2_ monoclinic form of vanadium dioxide. X-ray absorption also revealed that changes in this distance in tungsten-doped VO_2_ correlated with the amount of tungsten doped into the lattice and therefore the degree to which the transition temperature was depressed^35^. Thus this direction seems to be of significance in the structural phase transition. Investigation of any changes in these spacings occurring may therefore confirm the prediction of the computational approach.

### High Resolution X-ray Diffraction

**Figure 4:**
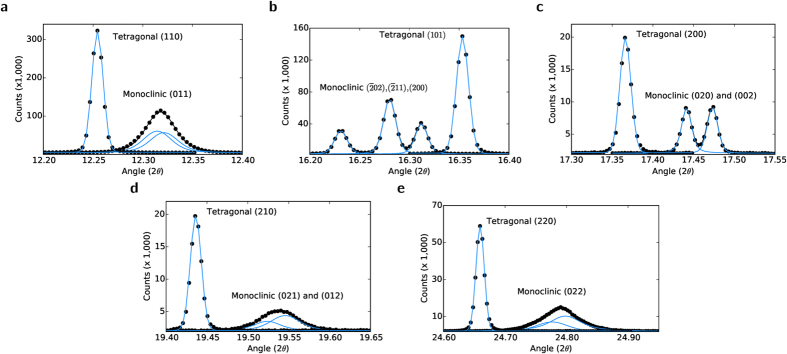
X-ray diffraction data and corresponding fits of (**a**) the monoclinic (011) and tetragonal (110) peaks, (**b**) the monoclinic 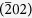, 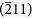 and (200) peaks and the corresponding tetragonal (101) peak, (**c**) the monoclinic (020) and (002) peaks and the corresponding tetragonal (200) peak, (**d**) the monoclinic (012) and (021) peaks and the corresponding tetragonal (210) peak, and (**e**) the monoclinic (022) and corresponding tetragonal (220) peaks.

To determine if there was any manifestation of the effects of this distortion in experimental data, high resolution X-ray diffraction was performed using the powder diffraction beamline at the Australian Synchrotron. Diffraction data of a sample of pure VO_2_ were recorded above and below the structural phase transition temperature of ~340 K, and Fig. 4 illustrates the most significant properties. Figure 4a contrasts the (110)_*T*_ and (011)_*M*_ peaks respectively (which correspond to the same planes, (see Fig. 1a,b). The data shows clearly that the transition from tetragonal to monoclinic results in slight splitting and considerable broadening of the peaks corresponding to the inter-vanadium distances along the [110]_*T*_ and 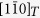 directions.

However, this broadening is not uniform. Figure 4b illustrates the (101)_*T*_ peak, and a triplet corresponding to the 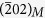, 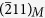, and (200)_*M*_ peaks (from low to high angle). In this case, the (101)_*T*_ and 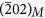 peaks correspond to the same distance, and despite a difference in amplitude, the peak shapes are very similar. Therefore, the data indicates that these distances do *not* experience any broadening as the structure transforms from tetragonal to monoclinic.

Figure 4c–e confirm this preferential orientation: Fig. 4c contrasts the (200)_*T*_ and (020)_*M*_,(002)_*M*_ peaks (again, which describe the same spacing) and similarly to Fig. 4b, no broadening is apparent. Figure 4d however, which contrasts the (210)_*T*_ and the (021)_*M*_,(012)_*M*_ peaks *does* exhibit the broadening observed in the (110)_*T*_ to (011)_*M*_ transition of Fig. 4a. Figure 4e provides confirmation of the data of Fig. 4a: it corresponds to the peaks at half the spacing: (220)_*T*_ and (022)_*M*_, and as before, broadening is observed.

Tables 1 and 2 presents the fit parameters of the tetragonal and monoclinic peaks of Fig. 4 respectively, which confirms this trend; the tetragonal peaks exhibit Gaussian broadening, however it is almost constant across the spectrum, varying only by a maximum of 10% from the mean. The monoclinic data on the other hand shows systematic variation. While the 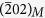, 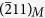, (200)_*M*_, (020)_*M*_ and (002)_*M*_ peaks exhibit roughly similar Gaussian broadening to the tetragonal peaks and a small amount of Lorentz broadening, the (011)_*M*_, (021)_*M*_, (012)_*M*_ and (022)_*M*_ are far broader. They exhibit Gaussian broadening which is approximately twice that of the tetragonal and other monoclinic peaks, and Lorentz broadening which is in some cases an order of magnitude larger, for example the (200) and (021) peaks.

Thus the data indicates that peaks of the form (0*xx*) or (0*xy*) experience significantly more broadening than other orientations. Such spacings describe distances with the same orientation as that of Fig. 3d; directed toward the neighboring vanadium chain. This disorder may be reconciled with the NEB data by taking into account the effects of defects and grain boundaries in the structure of the experimental sample. Figure 3d indicates that a distance is initially compressed, and subsequently extends. Figure 2 suggests that this compression costs energy. Thus, if structural defects are present which allow dissipation of this energy, the transformation to the monoclinic structure may be incomplete. Obviously individual grain boundaries will place limits on the extent of the propagation of this, and therefore it is possible that due to this, the strain broadening of the individual grains comprise a distribution which is anisotropic in nature.

**Figure 5:**
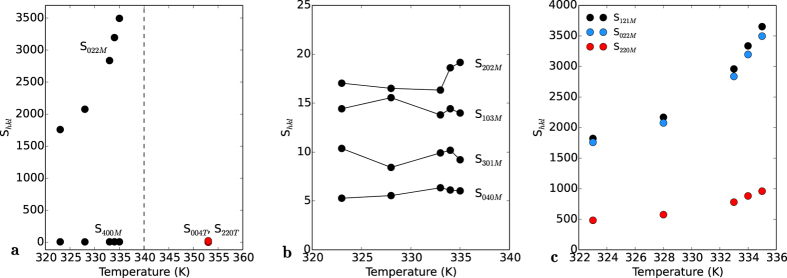
(**a**) Temperature-dependent comparison of strain broadening parameters extracted using the Stephens method. S_022*M*_ and S_400*M*_ correspond to the covariances of the *B* and *C* reciprocal axes and the variance of the *A* axis for the Monoclinic structure respectively and are plotted in black. S_220*T*_ and S_400*T*_ are the equivalent parameters for the Tetragonal structure, plotted in red. A clear divergence in the covariance of the Monoclinic *B* and *C* axes (S_022*M*_) is observed as the temperature approaches the critical point (~340 K, indicated by a dashed line). (**b**) Comparison of the Monoclinic Stephens strain parameters with no component along the Monoclinic **b**-axis and (**c**) Comparison of the S_121*M*_, S_022*M*_ and S_202*M*_ parameters, illustrating that while there is some correlation between broadening involcing the **a**- and **c**-axes, it is far smaller than the broadening involving both the **b**- and **c**-axes.

To investigate the possible manifestation of this, anisotropic strain broadening parameters were extracted using the Stephens method^37^. Figure 5a plots the magnitudes of the S_022_ and S_040_ contributions to the broadening for the M_1_ structure for five temperatures below the critical point, and contrasts them with the equivalent Tetragonal S_400_ and S_220_ contributions respectively (the mismatch in indices between the monoclinic and tetragonal parameters is due to the aforementioned different naming conventions of crystallographic axes in the cells, thus S_040*M*_ = S_400*T*_ and S_022*M*_ = S_220*T*_). The co-existence of the monoclinic and tetragonal structures near the critical temperature creates issues for fitting, and thus the data of Fig. 5 is limited to those points near the transition temperature which exhibited the best fitting parameters.

While the S_040*M*_ data is independent of temperature, and approximately equivalent in magnitude to the S_400*T*_ data, the S_022*M*_ data diverges as the temperature approaches the critical point. This contrasts sharply with the S_220*T*_ data which is almost *un-*correlated, as S_220*T*_ = −1.4, which is very close to zero. This data therefore indicates that as the critical temperature is approached from below, the contributions to peak broadening from variations in the *h* and *k* spacing become increasingly correlated, and the magnitudes of the variances increase rapidly. In other words, the broadening observed contains components along both the crystallographic **a** and **b** axes.

Figure 5b illustrates that the Stephens parameters corresponding to the only the **b**-axis (S_040_), and those with no contribution at all from the **b**-axis are low in magnitude, and show no temperature dependencies. Figure 5c illustrates that while there is a correlation between the monoclinic **a**- and **b**-axes in the behaviour of the S_220*M*_ parameter, it is dwarfed by the behaviour of the parameters in which contributions from the **b**- and **c**-axes are present: S_121*M*_ and S_022*M*_.

Figures 5a–c thus reveal that the diffraction data contains contributions to the broadening of the peaks which is anisotropic in nature, and that the most significant contributions are those of the S_022_ and S_121_ parameters, while the tetragonal structure shows no significant correlation in the equivalent parameters. This data is therefore in line with the data of Fig. 4, and Tables 1 and 2, and supports the hypothesis that the energy barrier between the structures corresponds to the compression and expansion of the characteristic distance of Fig. 3d.

The observed behaviour of the S_220_ data is in some respects not surprising, as when plotted as a function of temperature, it is basically an un-normalized temperature correlation function of the variances of the a* and b* axes. The divergence of this correlation at the T_*c*_ point is in line with critical behaviour expected at a phase transition, however, we do not attempt to explore this aspect in this work.

### Electronic Structure

What remains to be determined however, is the effect of these structural rearrangements on the electronic structure. There is little question that the electronic structures of the tetragonal and monoclinic forms are metallic and insulating respectively^29^, however of interest in this study is the behaviour of the band structure across this structural phase transition, in order to determine whether the structural and electronic phase transitions are intrinsically related, or in fact merely coincident. The next section explores this in detail.

**Figure 6:**
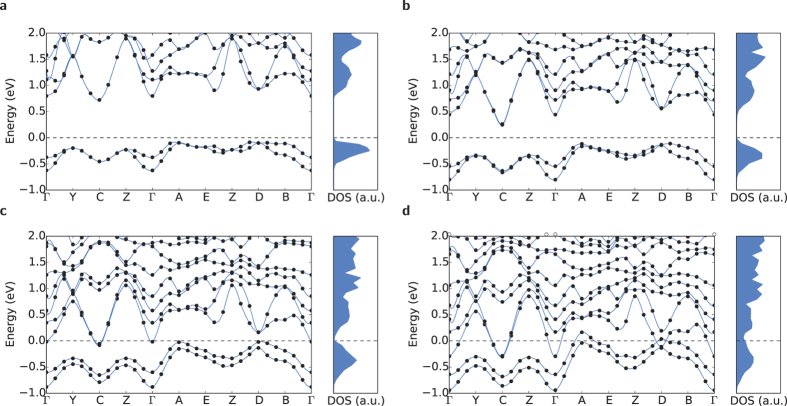
GW band eigenvalues (filled black circles fitted with blue splines to guide the eye, left panel) and electronic densities of states (filled blue curve, right panel) of (**a**) the M_1_ structure, (**b**) the “Step 1” structure, (**c**) the “Step 2” structure and (**d**) the “Step 3” structure. The Step numbers correspond to the total energy points in Fig. 2.

The band structures of the monoclinic ground state, and structures “Step 1”, “Step 2” and “Step 3” calculated using the GW approximation (black filled circles, fitted with blue splines) are presented in Fig. 6. The densities of states are plotted next to each band structure, on the same energy scale (blue filled curve). As expected, the monoclinic structure is insulating, with a band gap of ~0.70 eV, in excellent agreement with experiments (0.70 eV)^38^.

However as the structure transitions to the slightly more symmetric forms of Step 1 and Step 2 the densities of states indicate that the gap closes, and the structure becomes metallic. The corresponding band structures indicate that this occurs *via* two simultaneous mechanisms. The dispersions of the valence bands in the Γ→*A* direction suggests that in comparison to the band minima at Γ, the higher energy states near E_*F*_ are shifting upwards, most significantly near *A* and *D*. At the same time, the conduction band minima at Γ shift downwards. In structure “Step 2” the conduction and valence bands overlap (this was determined by inspection of the charge density), and the indirect gap closes. The band structure of Fig. 6c, when compared with the total energy data of Fig. 2, indicates that the electronic band gap destabilises before the top of the energy barrier is crested.

**Figure 7:**
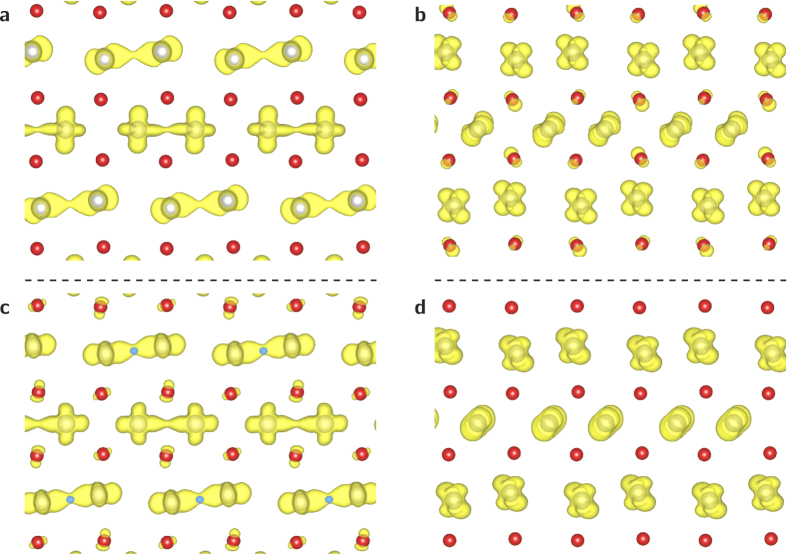
Charge densities in the 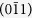 plane of (**a**) the highest valence band state at Γ, (**b**) the lowest conduction band state at Γ, (**c**) the highest valence band state at *D* and (**d**) the lowest conduction band state at *D*. The conduction and valence band states correspond to bonding and anti-bonding configurations respectively, and thus as the gap narrows between them across the transition (see Fig. 6) this indicates the bonding-anti bonding splitting is being reduced which results in the closing of the electronic band gap. Vanadium atoms are gray, while oxygen atoms are red.

We can gain a better idea of how the structural transitions are affecting these states by transforming them to real space charge densities and comparing them. Figure 7a,b present charge density isosurfaces of the valence and conduction band states at Γ in the 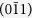 plane of the ground state monoclinic structure respectively, while Fig. 7c–d present charge density isosurfaces in the 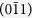 plane of the valence and lowest energy conduction band states at the *D* point of the M_1_ structure.

From comparison of Fig. 7a,b, it is obvious that the valence band state at Γ consists of shared charge density between the vanadium (grey spheres) atoms, while the conduction band state corresponds to isolated density on each vanadium atom. This suggests that the gap between the valence and conduction bands arises from bonding/antibonding splitting. The same story is repeated at *D*. The valence band state contains density linking the Peierls paired vanadium atoms, while the conduction band state consists again of isolated density on each vanadium atom. The band structure data of Fig. 6 indicate that as the structure transitions away from the M_1_ form and towards the tetragonal, the splitting between these states decreases considerably, with the eigenvalues at *D* crossing over by Step 3.

This charge density, combined with the eigenvalues, and the inter-vanadium spacing data of Fig. 3 indicate that as the structural phase transition progresses, starting from the M_1_ structure, the inter-vanadium distance of the Peierls pairs increases, destabilising bonding states with respect to anti-bonding states, narrowing the gap between the conduction and valence bands, until it disappears completely and the structure becomes metallic.

**Figure 8:**
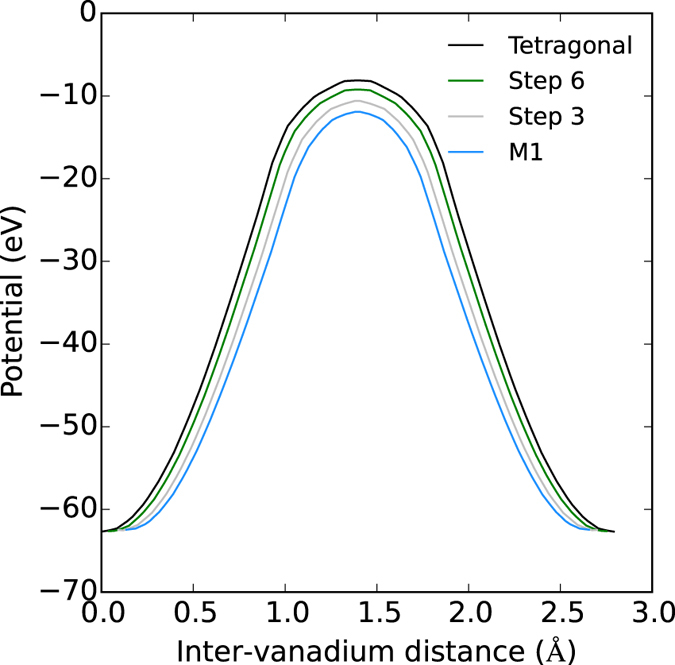
Total local potentials (eV) along a line segment connecting the Peierls paired vanadium atoms of the M_1_, Step 3, Step 6 and tetragonal structures. The inter-vanadium distances have been shifted such that the midpoints of each segment coincide, in order to align the potential barriers.

Figure 8 plots the value of the local potential along a line segment connecting the Peierls paired vanadium atoms of the M_1_, “Step 3”, “Step 6” and tetragonal structures, and from the data, it is obvious that as the inter-vanadium distance increases, the height of the potential barrier between the nuclei also increases. This increase will significantly affect the wavefunctions of the highest energy electrons which are obviously less tightly bound, resulting in less electron sharing between the paired atoms, consequently raising the energy of bonding configurations with respect to anti-bonding.

## Discussion

A more complete picture of the processes occurring across the VO_2_ structural/electronic phase transition can now be pieced together using the data presented. The barrier which separates the structures is a consequence of the need to compress the inter-vanadium spacing along the [011] and 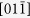 directions of the monoclinic structure in order to effect the structural phase transition. This mirrors the appearance of the M_2_ structure of VO_2_ upon the input of strain along the [110]_*T*_ direction^36^. The chains consist of a linear, Pe These displacements result in the destabilisation of the bond/anti-bonding splitting of the monoclinic structure, due to the increase in the potential barrier between the Peierls paired vanadium atoms. This results in the conduction and valence bands overlapping; an insulator-metal transition.

If the energy barrier is indeed a consequence of the atomic motion of Fig. 3d, then attempts to manipulate the transition temperature which involve modulation of the stress or strain along the [110]_*T*_ and 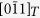 directions would produce the most significant effects. This is consistent with X-ray absorption studies^35^ which indicate that the depression of the transition temperature by tungsten-doping correlates with an increase in the V-V corner spacing. If the energy increase of Fig. 2 is a consequence of electronic repulsion, then increasing the V-V corner distances will lower the repulsion due to contraction as the increased internuclear separation will result in a lowering of the electrostatic potential between the atoms, reducing the barrier height.

## Methods

Variable temperature Synchrotron X-ray Powder Diffraction was conducted at the Australian Synchrotron Powder Diffraction Beamline. Samples were sealed in 0.3 mm borosilicate glass capillaries. Prior to data collection, the wavelength was set at 0.82732 Å using a Si (111) double crystal monochromator. The exact wavelength was refined using the NIST 660b LaB_6_ standard reference material. A Cyberstar hot-air blower was used to control the temperature to within 0.1–0.2 °C at each data collection temperature. Traces were recorded for 5 minutes at each of the two detector settings after the sample had reached the set point temperature and equilibrated for 10 minutes.

Quantitative Rietveld analysis was performed on the data using the Bruker TOPASTM V4.2 program to determine the weight percentage of phases present. Background signal was described using a Chebyshev polynomial linear interpolation function. A broad pseudo-Voight function was also used to model the background contribution form the capillary. Cell parameters, atom positions, (tightly constrained) isotropic thermal parameters, Gaussian and Lorentzian contributions to peak full widths at half maximum and scale factor were all refined.

The anisotropic broadening of the diffraction peaks observed in Fig. 4 was hypothesized to originate from one of two sources. Either there was some anisotropy in the crystallite shapes, leading to broadening of lines corresponding to directions in which fewer planes are stacked, or the crystal grains exhibit a distribution of residual strains originating from the transition from tetragonal to monoclinic. Thermal effects were deemed an unlikely origin, as refinements produced similar thermal parameters at all temperatures, and the anisotropic broadening observed is *smaller* at higher temperature. In addition, the Debye-Waller factor^39^,^40^ tends to reduce the scattered intensity, however in comparisons between the equivalent monoclinic and tetragonal peaks, such as (011)_*M*_ and (110)_*T*_ the peaks integrate to the same total intensity. Employing Jarvinen’s method^41^ to account for anisotropic broadening led to rather poor fits in comparison to those generated by the Stephens method^37^ for strain broadening, indicating that while some crystallite size ansiotropy may exist, the broadening is dominated by the strain distribution.

Strain analysis of the X-ray data was performed using the method developed by Stephens^37^, which is a phenomenological approach to determining the contributions to broadening induced by anisotropic variations in plane spacings. We repeat the central thesis of this approach here, but for a more complete treatment the reader is referred to the original work^37^.

The spacing of planes with Miller indices *hkl* is given by:


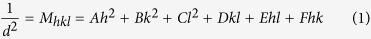


Re-labeling the metric parameters {*A*, …, *F*} as {*α*_*i*_} and assuming that they have Gaussian distributions characterised by a covariance matrix 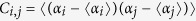, the variance of *M*_*hkl*_ can be written:


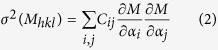


which since 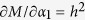, 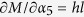 *etc.* can be re-written:


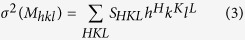


where from equations (1) and (2) the terms S_*HKL*_ are obviously defined for *H* + *K* + *L* = 4. The contribution from anisotropic strain broadening to the full-width-half-maximum (FWHM) of a diffraction line can be written using the Bragg equation and (4) as:


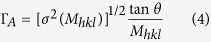


This Γ_*A*_ is combined with the usual parameters for Gaussian and Lorentizian line-widths to give expressions for anisotropically broadened line-shapes which are fitted to the experimental data, and the S_*hkl*_ are extracted from the fit. The Gaussian and Lorentzian broadening parameters of Tables 1 and 2 were extracted from fits to the individual peaks presented in the data of Fig. 4.

### Force calculations

The monoclinic^42^ and tetragonal^43^ structural parameters were input to DFT geometry relaxations using the VASP code^44^ and the Generalized Gradient Approximation to exchange and correlation of Perdew *et al*.^45^, on 6 × 6 × 6 and 8 × 8 × 6 Monkhorst-Pack^46^ k-space grids. The structures were then relaxed to their respective ground states using Methfessel and Paxton smearing^47^ and the conjugate gradient algorithm. Upon reaching the desired ground states, a 1 × 1 × 2 supercell of the tetragonal structure was constructed in order to have the same dimensions as the monoclinic form.

The Cartesian atomic positions of the tetragonal structure were then subtracted from those of the monoclinic structure, which generated vectors describing the movement of the atoms across the transition. Vectors describing the changes in unit cell dimensions were obtained in the same manner. These vectors were then divided such that 10 structures were generated, with the monoclinic structure being the first, and the tetragonal being the last and the intermediate structures are labelled “Step 1” to “Step 8”. The elastic band technique^34^ was then applied to these structures, in order to find the minimum energy path between them. The use of DFT to determine the total energies of Fig. 2 from the structures optimised by the elastic band method, rather than DFT+U or hybrid functionals stems from the requirement to maintain a consistent Hamiltonian for the calculation of the energies along the minimum energy path as the energy landscape, by definition, will be Hamiltonian dependent.

### Electronic Structure

The relaxed structural parameters were used as input to Density Functional Theory^44^,^48^ calculations on 6 × 6 × 6 Monkhorst-Pack k-space grids, again using the Generalized Gradient Approximation (GGA) approach to exchange and correlation of Perdew *et al*.^45^ with the Brillouin zone integration approach of Bloechl *et al*.^49^. Frequency-dependent GW calculations^50^ were performed using the implementation of Shishkin and Kresse^51^ in VASP^44^. The GW calculations were performed using a grid of 50 frequency points and an energy cutoff of 200 eV.

## Additional Information

**How to cite this article**: Booth, J. M. *et al*. Correlating the Energetics and Atomic Motions of the Metal-Insulator Transition of M_1_ Vanadium Dioxide. *Sci. Rep.* **6**, 26391; doi: 10.1038/srep26391 (2016).

## Figures and Tables

**Table 1:** Gaussian broadening parameters (σ) of the fits to the diffraction peaks of the tetragonal structure.

Plane	σ
(110)	5.7 × 10^−3^
(101)	6.1 × 10^−3^
(200)	6.8 × 10^−3^
(210)	6.7 × 10^−3^
(220)	6.7 × 10^−3^

**Table 2:** Gaussian (*σ*) and Lorentzian (Γ) broadening parameters of the Voigt fits to the diffraction peaks of the monoclinic structure.

Plane	*σ*	Γ
(011)	1.05 × 10^−2^	7.7 × 10^−3^
(011)	1.03 × 10^−2^	7.8 × 10^−3^
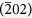	5.2 × 10^−3^	1.9 × 10^−3^
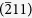	5.2 × 10^−3^	1.8 × 10^−3^
(200)	5.2 × 10^−3^	1.7 × 10^−3^
(020)	4.4 × 10^−3^	3.2 × 10^−3^
(002)	5.7 × 10^−3^	1.5 × 10^−3^
(021)	1.04 × 10^−2^	1.17 × 10^−2^
(012)	1.2 × 10^−2^	1.02 × 10^−2^
(022)	2.08 × 10^−2^	9.7 × 10^−3^
(022)	1.08 × 10^−2^	2.5 × 10^−2^

## References

[b1] Morin F. J. Oxides which show a metal-to-insulator transition at the Neel temperature. Phys. Rev. Lett. 3, 2–4 (1959).

[b2] Zylberstein A. & Mott N. F. Metal-insulator transition in vanadium dioxide. Phys. Rev. B 11, 4383–4395 (1975).

[b3] Eyert V. The metal-insulator transitions of VO_2_: A band theoretical approach. Ann. Phys. 11, 650–702 (2002).

[b4] Verleur H. W., Barker A. S. & Berglund C. N. Optical Properties of VO_2_ between 0.25 and 5 eV. Phys. Rev. 172, 788–798 (1968).

[b5] Cavalleri A. . Femtosecond Structural Dynamics in VO_2_ during an Ultrafast Solid-Solid Phase Transition. Phys. Rev. Lett. 87, 237401 (2001). 11736474 10.1103/PhysRevLett.87.237401

[b6] Goodenough J. B. The Two Components of the Crystallographic Transition in VO_2_. J. Solid State Chem. 3, 490–500 (1971).

[b7] Pouget J. P. . Dimerization of a linear Heisenberg chain in the insulating phases of V_1−*x*_Cr_*x*_O_2_. Phys. Rev. B 10, 1801–1815 (1974).

[b8] Wei J., Wang Z., Chen W. & Cobden D. H. New aspects of the metal-insulator transition in single-domain vanadium dioxide nanobeams. Nat. Nanotechnol. 4, 420–424 (2009). 19581893 10.1038/nnano.2009.141

[b9] Nakano M. . Collective bulk carrier delocalization driven by electrostatic surface charge accumulation. Nature 487, 459–62 (2012). 22837001 10.1038/nature11296

[b10] Wei J., Ji H., Guo W., Nevidomskyy A. H. & Natelson D. Hydrogen stabilization of metallic vanadium dioxide in single-crystal nanobeams. Nat. Nanotechnol. 7, 357–362 (2012).

[b11] Liu M. . Terahertz-field-induced insulator-to-metal transition in vanadium dioxide metamaterial. Nature 487, 345–8 (2012). 22801506 10.1038/nature11231

[b12] Park J. H. . Measurement of a solid-state triple point at the metal-insulator transition in VO_2_. Nature 500, 431–4 (2013). 23969461 10.1038/nature12425

[b13] Cao J. . Strain engineering and one-dimensional organization of metal-insulator domains in single-crystal vanadium dioxide beams. Nat. Nanotechnol. 4, 732–7 (2009). 19893528 10.1038/nnano.2009.266

[b14] Wu C., Feng F. & Xie Y. Design of vanadium oxide structures with controllable electrical properties for energy applications. Chem. Soc. Rev. 42, 5157 (2013). 23482986 10.1039/c3cs35508j

[b15] Wentzcovitch R. M., Schulz W. W. & Allen P. B. VO_2_: Peierls or Mott-Hubbard? A Vew from Band Theory. Phys. Rev. Lett. 72, 3389–3392 (1994). 10056186 10.1103/PhysRevLett.72.3389

[b16] Tomczak J. M., Aryasetiawan F. & Biermann S. Effective bandstructure in the insulating phase versus strong dynamical correlations in metallic VO2. Phys. Rev. B 78, 115103 (2008).

[b17] Biermann S., Poteryaev A., Lichtenstein A. & Georges A. Dynamical Singlets and Correlation-Assisted Peierls Transition in VO2. Phys. Rev. Lett. 94, 026404 (2005). 15698203 10.1103/PhysRevLett.94.026404

[b18] Tomczak J. M. & Biermann S. Effective band structure of correlated materials: the case of VO_2_. J. Phys. Condens. Matter 19, 365206 (2007). 21694152 10.1088/0953-8984/19/36/365206

[b19] Gatti M., Bruneval F., Olevano V. & Reining L. Understanding Correlations in Vanadium Dioxide from First Principles. Phys. Rev. Lett. 99, 266402 (2007). 18233592 10.1103/PhysRevLett.99.266402

[b20] Belozerov A. S., Korotin M. A., Anisimov V. I. & Poteryaev A. I. Monoclinic M_1_ phase of VO_2_: Mott-Hubbard versus band insulator. Phys. Rev. B 85, 045109 (2012).

[b21] Sohn J. I. . Surface-stress-induced Mott transition and nature of associated spatial phase transition in single crystalline VO_2_ nanowires. Nano Lett. 9, 3392–7 (2009). 19785429 10.1021/nl900841k

[b22] Jones A. C., Berweger S., Wei J., Cobden D. & Raschke M. B. Nano-optical investigations of the metal-insulator phase behavior of individual VO_2_ microcrystals. Nano Lett. 10, 1574–81 (2010). 20377237 10.1021/nl903765h

[b23] Aetukuri N. B. . Control of the metal-insulator transition in vanadium dioxide by modifying orbital occupancy. Nat. Phys. 9, 661–666 (2013).

[b24] Terauchi H. & Cohen J. B. Diffuse x-ray scattering due to the lattice instability near the metal-semiconductor transition in VO_2_. Phys. Rev. B 17, 2494–2496 (1978).

[b25] Gervais F. & Kress W. Lattice dynamics of oxides with rutile structure and instabilities at the metal-semiconductor phase transitions of NbO2 and VO2. Phys. Rev. B 31, 4809–4814 (1985). 10.1103/physrevb.31.4809 9936441

[b26] Cavalleri A., Dekorsy T., Chong H. H. W., Kieffer J. C. & Schoenlein R. W. Evidence for a structurally-driven insulator-to-metal transition in VO_2_: A view from the ultrafast timescale. Phys. Rev. B 70, 161102 (2004).

[b27] Kim H.-T. . Monoclinic and Correlated Metal Phase in VO2 as Evidence of the Mott Transition: Coherent Phonon Analysis. Phys. Rev. Lett. 97, 266401 (2006). 17280436 10.1103/PhysRevLett.97.266401

[b28] Wall S. . Ultrafast changes in lattice symmetry probed by coherent phonons. Nat. Commun. 3, 721 (2012). 22395612 10.1038/ncomms1719

[b29] Zheng H. & Wagner L. K. Computation of the Correlated Metal-Insulator Transition in Vanadium Dioxide from First Principles. Phys. Rev. Lett. 114, 176401 (2015). 25978243 10.1103/PhysRevLett.114.176401

[b30] Laad M. S., Craco L. & Müller-Hartmann E. Metal-insulator transition in rutile-based VO_2_. Phys. Rev. B 73, 195120 (2006).

[b31] Chen S., Liu J., Luo H. & Gao Y. Calculation Evidence of Staged Mott and Peierls Transitions in VO_2_ Revealed by Mapping Reduced-Dimension Potential Energy Surface. J. Phys. Chem. Lett. 6, 3650–3656 (2015). 26722737 10.1021/acs.jpclett.5b01376

[b32] Anisimov V. I., Zaanen J. & Andersen O. K. Band theory and Mott insulators: Hubbard U instead of Stoner I. Phys. Rev. B 44, 943–954 (1991). 10.1103/physrevb.44.943 9999600

[b33] Booth J. M. & Casey P. S. Production of VO_2_ M_1_ and M_2_ nanoparticles and composites and the influence of the substrate on the structural phase transition. ACS Appl. Mater. Interfaces 1, 1899–905 (2009). 20355812 10.1021/am900322b

[b34] Henkelman G., Uberuaga B. P. & Jonsson H. A climbing image nudged elastic band method for finding saddle points and minimum energy paths. J. Chem. Phys. 113, 9901–9904 (2000).

[b35] Booth J. M. & Casey P. S. Anisotropic structure deformation in the VO_2_ metal-insulator transition. Phys. Rev. Lett. 103, 1–4 (2009). 10.1103/PhysRevLett.103.086402 19792742

[b36] Pouget J. P., Launois H., D’Haenens J. P., Merenda P. & Rice, Tim, M. Electron Localization Induced by Uniaxial Stress in Pure VO_2_. Phys. Rev. Lett. 35, 873–875 (1975).

[b37] Stephens P. W. Phenomenological model of anisotropic peak broadening in powder diffraction. J. Appl. Crystallogr. 32, 281–289 (1999).

[b38] Shin S. . Vacuum-ultraviolet reflectance and photoemission study of the metal-insulator phase transitions in VO_2_, V_6_O_13_, and V_2_O_3_. Phys. Rev. B 41, 4993–5009 (1990). 10.1103/physrevb.41.4993 9994356

[b39] Debye P. Interferenz von Röntgenstrahlen und Wärmebewegung. Ann. Phys. 348, 49–92 (1913).

[b40] Waller I. Zur Frage der Einwirkung der Wärmebewegung auf die Interferenz von Röntgenstrahlen. Zeitschrift für Phys. 17, 398–408 (1923).

[b41] Jarvninen M. Application Of Symmetrized Harmonics Expansion To Correction Of The Preferred Orientation Effect. J. Appl. Crystallogr. 26, 525–531 (1993).

[b42] Andersson G. Studies on Vanadium Oxides. I. Phase Analysis. Acta Chem. Scand. 8, 1599–1606 (1954).

[b43] Marezio M., McWhan D. B., Remeika J. P. & Dernier P. D. Structural Aspects of the Metal-Insulator Transitions in Cr-Doped VO_2_. Phys. Rev. B 91, 2541–2551 (1971).

[b44] Kresse G. & Furthmüller J. Efficient iterative schemes for ab initio total-energy calculations using a plane-wave basis set. Phys. Rev. B 54, 11169–11186 (1996). 10.1103/physrevb.54.11169 9984901

[b45] Perdew J. P., Burke K. & Ernzerhof M. Generalized Gradient Approximation Made Simple. Phys. Rev. Lett. 77, 3865–3868 (1996). 10062328 10.1103/PhysRevLett.77.3865

[b46] Monkhorst H. J. & Pack J. D. Special points for Brillouin-zone integrations. Phys. Rev. B 13, 5188–5192 (1976).

[b47] Methfessel M. & Paxton A. T. High-precision sampling for Brillouin-zone integration in metals. Phys. Rev. B 40, 3616–3621 (1989). 10.1103/physrevb.40.3616 9992329

[b48] Kohn W. & Sham L. J. Self-consistent equations including exchange and correlation effects. Phys. Rev. 140, 1133–1138 (1965).

[b49] Blochl P. E., Jepsen O. & Andersen O. K. Improved tetrahedron method for Brillouin-zone integrations. Phys. Rev. B 49, 16223–16233 (1994). 10.1103/physrevb.49.16223 10010769

[b50] Hedin L. New Method for Calculating the One-Particle Green’s Function with Application to the Electron-Gas Problem. Phys. Rev. 139, 796–823 (1965).

[b51] Shishkin M. & Kresse G. Implementation and performance of the frequency-dependent GW method within the PAW framework. Phys. Rev. B 74, 035101 (2006).

